# Psychological features of systemic sclerosis: results from an observational study

**DOI:** 10.3389/fmed.2024.1473587

**Published:** 2024-12-06

**Authors:** Sara Romanazzo, Caroline Rometsch, Alessia Marangoni, Serena Guiducci, Fiammetta Cosci

**Affiliations:** ^1^Department of Health Sciences, University of Florence, Florence, Italy; ^2^Department of Experimental and Clinical Medicine, University of Florence, Florence, Italy; ^3^Rheumatology Unit, Department of Experimental and Clinical Medicine, University of Florence, Florence, Italy; ^4^Department of Psychiatry and Neuropsychology, Maastricht University, Maastricht, Netherlands

**Keywords:** systemic sclerosis, psychological distress, wellbeing, paranoid ideation, interpersonal relationship

## Abstract

**Objectives:**

(a) Assessing mental disorders, psychological distress, psychological wellbeing in patients with systemic sclerosis (SSc); (b) identifying psychological features independently contributing to the status of having the diagnosis of SSc.

**Methods:**

Two hundred SSc outpatients were compared with 100 healthy subjects. Mental disorders were assessed via the Mini International Neuropsychiatric Interview (MINI). Self-reported rating scales were administered: Health Assessment Questionnaire Disability Index (HAQ-DI), Symptom Checklist-90-Revised (SCL-90-R), Psychological Well Being scales (PWB). General linear models allowed to verify which psychological feature would individually make unique contributions to overall status of having the diagnosis of SSc.

**Results:**

Major depressive episode/disorder, panic disorder were more prevalent among patients with SSc (*p* < 0.05); SCL-90-R somatization and depression were more severe (*p* < 0.05) in SSc; PWB personal growth, positive relationships with others, purposes in life were poorer (*p* < 0.05) in patients with SSc if compared to healthy controls. The final general linear model, accounting for 20.4% of variance, showed that having the diagnosis of SSc was associated to lower SCL-90-R paranoid ideation and poorer PWB relationships with others.

**Conclusion:**

SSc showed to present psychological features in need of assessment since some of them individually made unique contributions to overall status of having the SSc diagnosis.

## 1 Introduction

Systemic sclerosis (SSc) is a rare chronic autoimmune rheumatic disease characterized by fibrosis of the skin (which becomes thicker and rigid) and internal organs (which interfere with their functioning) and vasculopathy (particularly evident from an aesthetic point of view as fingers’ telangiectasia) (1). It has high morbidity and mortality (2). It benefits from pharmacological interventions in terms of decrease of symptoms severity and improvement of quality of life (3) since recovery is not currently possible. Overall, the severity of non-lethal physical complications is substantial and is likely to be increased by psychological and mental complications (4). Mental disorders, mainly depressive and anxiety disorders, showed to be more prevalent among patients with SSc than healthy subjects (5), with a rate of 6% for panic disorder and of 19% for major depressive disorder (6). Psychological distress is also rather common with particular reference to sadness, anhedonia, hopelessness, loss of energy (7–12), and anxiety (13). Psychological distress contributes to the physical and emotional impact of SSc (14), particularly in terms of quality-of-life deterioration (15). Overall, mental disorders and psychological distress seem to be an issue in patients with SSc, even though they have not been largely investigated.

Positive psychological characteristics, such as resilience, psychological wellbeing, optimism, self-efficacy, among the others, showed to play a buffering or protective role in patients with medical diseases. For instance, they were increasingly associated with improved outcomes in diabetes (16). The evidence available on such features in SSc is extremely sparse. Resilience showed to be negatively correlated with anxiety and depression (17) and with sense of coherence (14). Psychological wellbeing was found poorer in SSc than in polymyalgia rheumatica, rheumatoid arthritis, systemic lupus erythematosus, primary Sjögren’s syndrome, or idiopathic inflammatory myositis (18). Psychological interventions specifically aimed at improving and empowering psychological wellbeing showed clinical benefits in patients with SSc if compared to treatment as usual (19). Thus, positive psychological features seem to be an issue worthy of investigation in patients with SSc, also because they might represent aspects which contribute to the overall distress due to the disease and possible targets of intervention.

In the present study, mental disorders, psychological distress, and wellbeing were investigated in patients with SSc and compared with healthy subjects. The aim was to have more insight on the occurrence of mental disorders and on the level of intensity of specific psychological features, namely distress and wellbeing, in patients with a diagnosis of SSc. The present study also aimed to test whether some of the psychological features investigated would individually make unique contribution to overall status of having a diagnosis of SSc.

## 2 Materials and methods

### 2.1 Participants and procedure

Two hundred participants with SSc were enrolled among outpatients at the Scleroderma Unit of Rheumatology of the University Hospital Careggi (Florence, Italy) from June 2020 to October 2022. The following inclusion criteria were applied: (a) age ≥ 18 years of age; (b) diagnosis of systemic sclerosis according to the 2013 American College of Rheumatology and the European League Against Rheumatism (ACR/EULAR) classification criteria (20). Exclusion criteria were: (a) changes in drug therapy within the past 3 months; (b) any other condition that might alter patient’s ability to follow the study procedures (e.g., cognitive impairment) based on clinicians’ evaluation.

SSc patients were compared to healthy subjects (*n* = 100) recruited from the general population of the same geographic area in the same period of time. The following inclusion criteria were applied: (a) age ≥ 18 years of age; (b) self-referred absence of chronic medical disease. The only exclusion criterion was having a self-reported condition that might alter the patient’s ability to follow the study procedures (e.g., cognitive impairment).

The two groups were matched for sex and age (ratio 2:1).

All participants provided and signed a written informed consent including a privacy protection disclaimer. The study protocol was approved by the Regional Ethical Committee for Clinical Experimentation of the Tuscan Region (protocol code: WBTinSSC, date: 25.02.2020).

### 2.2 Assessment instruments

Mental disorders were diagnosed via the Mini International Neuropsychiatric Interview (MINI) (21). Psychological distress was gauged using the Symptom Checklist-90-Revised (SCL-90-R) (22). Psychological wellbeing was assessed through the Psychological Well Being scales (PWB) (23). In addition, functional disability due to SSc was measured via the Health Assessment Questionnaire Disability Index (HAQ-DI) (24).

The Mini International Neuropsychiatric Interview (21) is a brief structured interview allowing to diagnose 17 mental disorders according to the Diagnostic and Statistical Manual of mental disorders (DSM) and the International Classification of Diseases (ICD). It covers around 120 items with binary *yes/no* answers which can be used to determine whether diagnostic criteria for a disorder are met. With an assessment time of approximately 15–30 min, it is a short and accurate instrument with high reliability and validity (25, 26).

The Symptom Checklist-90-Revised (22) is a 90-item self-report multidimensional symptom inventory designed to assess psychological symptoms and distress. Each item is rated on a 5-point Likert scale (from 0 = not at all to 4 = extremely) assessing nine psychological dimensions: somatization, obsessive-compulsive, interpersonal sensitivity, depression, anxiety, hostility, phobic anxiety, paranoid ideation, psychoticism. Higher scores on each scale indicate higher level of the specific psychological manifestation. A global index of distress, the Global Severity Index (GSI), aggregates the 90 items to gauge an overall psychological distress. Higher score on the GSI indicates higher level of psychopathological distress (27). The SCL-90-R showed to be a reliable instrument (28).

The Psychological Well Being scales (23) assess psychological wellbeing through 84 items that cover six domains: autonomy, environmental mastery, personal growth, positive relationships with others, purposes in life, self-acceptance. Responses are rated on a 6-point Likert scale ranging from 1 (strongly disagree) to 6 (strongly agree), yielding a total score spectrum of 84–504 where higher scores denote a greater level of wellbeing. The instrument has shown good reliability (29).

The Health Assessment Questionnaire Disability Index (24) is a widely used assessment tool for measuring functional disability. It includes 20 items across 8 domains such as dressing, rising, eating, walking, hygiene, reach, grip, and usual activities, scored from 0 to 3, where 0 indicates “no difficulty” and 3 indicates “unable to do.” The scores of those domains are averaged resulting in a disability index score which ranges from 0 to 3. A score 0 or 1 suggests mild to moderate difficulties; scores from 1 to 2 indicates moderate to severe disabilities; scores from 2 to 3 suggests severe or very severe disabilities (30). HAQ-DI has shown good validity and reliability (31).

### 2.3 Statistical analysis

Descriptive statistics were calculated regarding baseline demographic and clinical variables. For the categorical variables (e.g., sex), percentages were calculated. For continuous ones (e.g., age), mean and standard deviation were calculated.

The SSc and the healthy control groups were compared via the chi-square (χ2) test in case of categorial variables (i.e., MINI diagnosis) and via the Mann–Whitney U test in case of non-normally distributed continuous variables (SCL-90-R and PWB scales). These statistical analyses were performed using IBM SPSS Statistics, version 26. A *p* ≤ 0.05 was considered statistically significant.

In order to test whether some of the psychological features investigated would individually make unique contribution to overall status of having a diagnosis of SSc, general linear models (GLMs) were performed (32) with manual backward and stepwise exclusion procedure since the literature does not suggest an *a priori* ranking of predictors of SSc or healthy status. The final model targets the one having the highest adjusted sum of squares and *F* values (33). The approach allows the data to determine a ranking of predicting variables based on their respective contributions to the overall variance. The GLM analyses were performed without adjusting for sex and age since the two groups were matched for such variables. Multicollinearity was assessed using the Variance Inflation Factor (VIF) and tolerance values, calculated with the vif() function from the *car* package in RStudio. Tolerance values were derived as the inverse of VIF (1/VIF). Following standard guidelines (34), VIF values below 5 and tolerance values above 0.2 are considered acceptable. In this model, all predictors met these criteria, with VIF values ranging from 1.24 to 5.17 and tolerance values from 0.19 to 0.80, indicating no multicollinearity. No outliers were found in the regression models. No participant’s data had associated studentized residual larger than ±3.0 or Cook’s distance >1 (35). These statistical analyses were conducted in RStudio version 4.3.0. The *MASS* package (36), which is based on the stepwise Akaike information criterion selection process, was used. HAQ total score, MINI, PWB, and SCL-90-R subscale scores were used as predictors. A basic summary was added including coefficients, standard errors, z-values, 95% confidence intervals (95%CI), odds ratios, degrees of freedom, McFadden’s pseudo R-squared, and the adjusted McFadden’s pseudo R-squared, to test the goodness-of-fit for logistic regression models.

## 3 Results

### 3.1 Descriptive variables

A total of 200 patients with a diagnosis of SSc (response rate: 73%) and 100 healthy subjects (response rate: 67%) was analyzed. Participants of the matched samples were mainly females (91%) with a mean age of 58.88 ± 13.27 years among patients with SSc and a mean age of 58.95 ± 13.32 years among healthy subjects (range 21–84) (see Table 1). The two groups did not differ for civil status while patients with SSc had lower education than healthy controls (χ^2^_(4)_ = 13.31, *p* = 0.010) and were less employed (χ^2^_(3)_ = 8.82, *p* = 0.032) (see Table 1).

**Table 1:** Descriptives.

	SSc patients	Healthy subjects	
	(*n* = 200)	(*n* = 100)	
	Mean ± SD	Mean ± SD	*p*
Age	58.88 ± 13.27	58.95 ± 13.32	0.959

	*n* (%)	*n* (%)	*p*
Sex, females	182 (91%)	91 (91%)	1.000
Living area			
Urban	113 (56.5%)	78 (78%)	0.000
Rural	87 (43.5%)	22 (22%)	
Marital status			
Married	132 (66.0%)	71 (71.0%)	0.558
Divorced/Separated	22 (11.0%)	10 (10.0%)	
Widowed	20 (10.0%)	8 (8.0%)	
Single	26 (13.0%)	11 (11.0%)	
Education			
Primary school	14 (7.0%)	4 (4.0%)	0.010
Secondary school	61 (30.5%)	19 (19.0%)	
High school	80 (40.0%)	48 (48.0%)	
University	45 (22.5%)	29 (29.0%)	
Working activity			
Yes	82 (41%)	58 (58.0%)	0.032
No	118 (59.0%)	42 (42.0%)	

Patients with SSc (*n* = 200) vs. healthy subjects (*n* = 100). Total numbers are presented, percentages in brackets. SSc, systemic sclerosis.

### 3.2 Clinical variables

Comorbid medical diseases were described in Table 2. Patients with SSc had more frequently comorbid physical disease than controls (*n* = 77; 38.5% vs. *n* = 16; 16%; *p* < 0.001).

**Table 2:** Physical comorbidities in patients with SSc (*n* = 200) and in healthy subjects (*n* = 100).

	SSc patients	Healthy subjects
	(*n* = 200)	(*n* = 100)
	*n* (%)	*n* (%)
Hypothyroidism	41 (20.5%)	10 (10.0%)
Gastroesophageal reflux	10 (5.0%)	0 (0.0%)
Rheumatoid arthritis	8 (4.0%)	0 (0.0%)
Osteoporosis	8 (4.0%)	2 (2.0%)
Sjögren’s syndrome	7 (3.5%)	0 (0.0%)
Arterial hypertension	5 (2.5%)	0 (0.0%)
Fibromyalgia	4 (2.0%)	0 (0.0%)
Hashimoto’s thyroiditis	3 (1.5%)	1 (1.0%)
Hiatal hernia	3 (1.5%)	1 (1.0%)
Diabetes	3 (1.5%)	2 (2.0%)
Migraine	2 (1.0%)	0 (0.0%)
Psoriatic arthritis	2 (1.0%)	0 (0.0%)
Psoriasis	1 (0.5%)	0 (0.0%)
Spondylarthritis	1 (0.5%)	0 (0.0%)
Myositis	1 (0.5%)	0 (0.0%)
Glaucoma	1 (0.5%)	0 (0.0%)
Mitral valve prolapse	1 (0.5%)	0 (0.0%)
Cardiomyopathy	1 (0.5%)	0 (0.0%)
Tricuspid insufficiency	1 (0.5%)	0 (0.0%)
Heart failure	1 (0.5%)	0 (0.0%)
Coronary artery disease	1 (0.5%)	0 (0.0%)
Biliary cirrhosis	1 (0.5%)	0 (0.0%)
Hepatic steatosis	1 (0.5%)	0 (0.0%)
Ulcerative colitis	1 (0.5%)	0 (0.0%)
Myelofibrosis	1 (0.5%)	0 (0.0%)
Neurosarcoidosis	1 (0.5%)	0 (0.0%)
Epicondylitis	0 (0.0%)	1 (0.5%)
Atopic dermatitis	0 (0.0%)	1 (0.5%)
Allergic asthma	0 (0.0%)	1 (0.5%)

Total numbers are presented, percentages in brackets. SSc, systemic sclerosis.

Sixty-five (32.2%) patients with SSc were diagnosed with a mental disorder vs. 21 (21.0%) healthy subjects (χ^2^_(1)_ = 4.31, *p* = 0.038). The following diagnoses were more prevalent among patients than among healthy subjects: major depressive episode, major depressive disorder, panic disorder (see Table 3).

**Table 3:** DSM-5 mental disorders.

	SSc patients	Healthy subjects		
	(*n* = 200)	(*n* = 100)		
	*n* (%)	*n* (%)	χ2	*p*
DSM-5 diagnoses				
Major Depressive Episode	54 (27.0%)	16 (16.0%)	4.51	0.034
Major Depressive Disorder	52 (26.0%)	14 (14.0%)	5.59	0.018
Suicidal Ideation	1 (0.5%)	1 (1.0%)	0.25	0.616
Panic Disorder	23 (11.5%)	4 (4.0%)	4.58	0.032
Agoraphobia	5 (2.5%)	1 (1.0%)	0.77	0.382
Social Anxiety Disorder	1 (0.5%)	0 (0.0%)	0.50	0.479
Generalized Anxiety Disorder	9 (4.5%)	3 (3%)	0.39	0.532
Post-traumatic Stress Disorder	2 (1.0%)	1 (1.0%)	0.00	1.00
Obsessive Compulsive Disorder	1 (0.5%)	0 (0.0%)	0.50	0.479
Bipolar Disorder	1 (0.5%)	1 (1.0%)	0.50	0.479
Anorexia Nervosa	1 (0.5%)	0 (0.0%)	0.50	0.479

Patients with SSc (*n* = 200) vs. healthy subjects (*n* = 100). Total numbers are presented, percentages in brackets. SSc, systemic sclerosis; DSM-5, diagnostic and statistical manual of mental disorders, fifth edition.

SCL-90-R somatization and depression scores were higher among patients than healthy subjects while PWB personal growth, positive relationships with others, and purposes in life were lower in patients with SSc than in healthy subjects (see Table 4). As expected, the mean HAQ-DI total score was 0.58 (SD = 0.60) among patients with SSc and 0.11 (SD = 0.34) in healthy subjects (*p* ≤ 0.001).

**Table 4:** Symptom checklist-90-revised (SCL-90-R) subscale scores, global severity index score, and psychological well being scales (PWB) scale scores.

	SSc patients	Healthy subjects	
	(*n* = 200)	(*n* = 100)	
	Mean (SD)	Mean (SD)	*p*
SCL-90-R subscales			
Somatization	0.77 (0.64)	0.46 (0.40)	≤0.001
Obsessive-compulsive	0.59 (0.57)	0.54 (0.49)	0.612
Interpersonal sensibility	0.35 (0.41)	0.37 (0.46)	0.809
Depression	0.57 (0.54)	0.45 (0.49)	0.024
Anxiety	0.42 (0.48)	0.35 (0.37)	0.390
Hostility	0.26 (0.38)	0.27 (0.40)	0.835
Phobic anxiety	0.19 (0.40)	0.12 (0.25)	0.143
Paranoid ideation	0.37 (0.44)	0.43 (0.46)	0.252
Psychoticism	0.22 (0.34)	0.18 (0.29)	0.315
**GSI**	0.46 (0.40)	0.38 (0.33)	0.113
PWB scales			
Autonomy	63.51 (10.82)	64.95 (8.88)	0.309
Environmental mastery	60.71 (11.01)	62.95 (10.31)	0.096
Personal growth	60.48 (9.75)	63.53 (8.08)	0.007
Positive relationships with others	63.73 (11.79)	66.68 (9.73)	0.024
Purposes in life	61.13 (10.49)	63.65 (9.96)	0.043
Self-acceptance	60.56 (12.45)	63.48 (10.9)	0.072

Patients with SSc (*n* = 200) vs. healthy subjects (*n* = 100). Mean scores and standard deviation (SD) are presented. SSc, systemic sclerosis; SCL-90-R, Symptom Checklist-90-Revised; GSI, Global Severity Index; PWB, Psychological Well Being scales; SSc, systemic sclerosis.

### 3.3 Psychological features individually making contribution to the SSc status

GLMs were conducted to test whether some of the psychological features under study would individually make unique contribution to overall status of having a diagnosis of SSc (Supplementary material S1). The final model (Model 15) accounted for 20.4% of variance and identified that having a diagnosis of SSc was associated with greater HAQ-DI disability (OR: 26.056, 95%CI: 9.540–85.255, *p* < 0.001), as expected, lower SCL-90-R paranoid ideation (OR: 0.338, 95%CI: 0.163–0.677, *p* = 0.003) and poorer PWB relationships with others (OR: 0.969, 95%CI: 0.941–0.996, *p* = 0.029) (*F*_(296)_∣SSc diagnosis∣HAQ,PWB,SCL) = 3.260 × HAQ − 0.031 × PWB − 1.085 × SCL, *p* < 0.001 (Table 5).

**Table 5:** Stepwise multiple regression analysis with health status (i.e., belonging to the systemic sclerosis patient group vs. healthy group) as dependent variable, final model.

	Dependent variables	df	β	*SE*	Z value	OR	95% LCI	95% UCI	*p*	*R* ^2^	*Adjusted R* ^2^
Model 15	HAQ-DI total score	296	3.260	0.556	5.856	26.056	9.540	85.255	<0.001	0.204	0.183
	PWB positive relationships with others	296	−0.032	0.014	−2.188	0.969	0.941	0.996	0.029		
	SCL-90-R paranoid ideation	296	−1.085	0.361	−3.000	0.338	0.163	0.677	0.003		

β, regression coefficient; SE, standard error; Z, ratio of the estimate to its standard error; OR, odds ratio; 95%LCI, lower value of the 95% confidence interval; 95%UCI, upper value of the 95% confidence interval; *R*^2^, McFaden Pseudo R; HAQ-DI, Health Assessment Questionnaire Disability Index; PWB, Psychological Well Being Scales; SCL-90-R, Symptom Checklist-90-Revised.

## 4 Discussion

The present study aimed at: (a) having more insight on mental disorders prevalence and levels of psychological distress and wellbeing in patients with a diagnosis of SSc; (b) testing whether some of the psychological features investigated would individually make unique contribution to the overall status of having a diagnosis of SSc. The main results showed that major depressive episode, major depressive disorder, and panic disorder were more prevalent among patients with SSc; SCL-90-R somatization and depression were more severe in the SSc group; PWB personal growth, positive relationships with others, purposes in life were poorer in patients with SSc than in healthy controls. The final general linear model, accounting for 20.4% of variance, identified that having the diagnosis of SSc was associated with lower SCL-90-R paranoid ideation and poorer PWB relationships with others.

The present work confirms that major depression is the most prevalent mental disorder in patients with SSc (4, 6, 37). It also confirms that anxiety disorders are highly represented. In the present study, panic disorder had the highest prevalence, followed by generalized anxiety disorder, which is consistent with Baubet et al. (6) but not with Jha et al. (4), who found generalized anxiety disorder more represented than panic. Overall, we may say that patients with SSc are at risk of presenting an anxious core, and this is particularly true if we consider that generalized anxiety and panic disorders have been described as two different stages of the same mental disorder (named panic disorder) according to the staging model, which allows to observe the longitudinal development of a mental disorder (38).

The high occurrence of depressive and anxious disorders in patients with SSc might be due to the unpredictable and progressive course of the medical disease which, together with an increasing functional disability, chronic pain, fatigue, body-image distress, and limited treatment options (39), expose patients to increasingly challenging situations, which can manifest themselves in a mental disorder. In this vein, patients with SSc also had more severe somatization and depressive symptoms, according to the SCL-90-R, which is consistent with previous studies (14, 40).

The most compromised psychological wellbeing dimensions in patients with SSc were personal growth, positive relationships with others, and purposes in life. This is the first time that specific dimensions, rather than an overall measure of psychological wellbeing, were assessed in systemic sclerosis and in comparison to healthy subjects. Of course, having a disease such as SSc can explain why these patients have an impaired subjective perception of their own personal growth and purposes in life, a great uncertainty on the future and on the future level of physical disability. In addition, the aesthetic impact of the disease (mainly due to facial amimia, consequent to skin fibrosis, and telangiectasia) may trigger a body-image distress (39) which can be a barrier to a satisfactory social life and intimacy (not only romantic) with people.

The final general linear model, accounting for 20.4% of variance, identified that having the diagnosis of SSc was associated with lower SCL-90-R paranoid ideation and poorer PWB relationships with others. The result regarding paranoid ideation seems to be contradicting in the limited literature available. One study found more severe SCL-90-R paranoid ideation in patients with SSc than in healthy subjects (41) while another study found no difference (14). To be noted that in Angelopoulos et al. (41) only females were enrolled and the sample size was lower than in Hyphantis et al. (14). These methodological differences may explain the contradicting results mentioned. The fact that we found paranoid ideation lower in patients than in healthy subjects is in any case in need of an explanation, not being consistent with the literature. It has been shown in medically ill patients that more altered physical conditions are associated with more altered mental status (42). We can thus hypothesize that the outpatient sample enrolled in the present study, who came from a tertiary level facility rather than a tertiary or secondary level facility as that enrolled by Angelopoulos et al. (41) and Hyphantis et al. (14), might have an overall better health condition. This also because we enrolled our sample about 15 years after Angelopoulos et al. (41) and Hyphantis et al. (14), which means that we currently have treatment options more effective than in the past in improving SSc symptoms. Indeed, the level of functional disability (HAQ-DI) due to systemic sclerosis in our sample was rather low (43–45).

Poor relationships with others also turned out to be distinctive of the SSc status. This specific psychological wellbeing dimension reflects the ability to establish affectionate, trusting, deep interpersonal relationships as well as the ability to demonstrate strong empathy and concern for the wellbeing of others (46). As mentioned above, it seems reasonable that the aesthetic impact of SSc as well as the distress of having such a disease might interfere with social functioning. Indeed, a poor psychosocial adjustment to illness in SSc patients was found to be related to dissatisfaction with social support (47). Moreover, limitations in private interpersonal relationships were reported by 41.2% of patients with SSc hand/feet/joint involvement, 42.9% of those with pulmonary fibrosis, and 46.3% of those with both complications (48). In addition, 48.6% of patients with SSc reported that the chronic disease changed their family social life (48). A breakdown in intimacy with partners on sexual/romantic relationship as well as social isolation and loss of friendships, particularly due to the unpredictability and fluctuating nature of SSc which make it difficult to plan activities with others, were also described (49).

The limitations of the present study include: (a) a monocentric design; future research could improve the robustness of the results ensuring that they are applicable to broader populations by using a multicenter approach thus involving a larger geographically heterogeneous sample; (b) the voluntary nature of participation, which may lead to selection bias as participants may have had higher motivation and a more stable health condition, which is also suggested by the low prevalence of mental comorbidity and by the low level of severity of paranoid ideation. This selection bias might affect the generalizability of results; (c) the sample size was not evenly distributed and limited, however the comparability of the data collected in patients with those collected in healthy controls was ensured by the matching procedure and SSc is a rare condition, thus such sample size seems adequate to increase knowledge on the topic; (d) the control group had lower co-occurring chronic conditions than the SSc group. This may introduce confounders and undermine the validity of the comparison between the two groups, as other chronic conditions may independently contribute to psychological issues. For the control group to serve as a more valid comparison, it should be matching the patients’ group except for the exposure, which is SSc in this case. However, this procedure would introduce other possible biases since SSc comorbid conditions can be diverse, such heterogeneity (represented both in the patients’ and in the controls’ group) might be a confounder as well. In addition, the use of healthy subjects as comparators is considered a possible, even though not the best, methodological option; (e) we used a cross-sectional design which captures data at a single point in time. A longitudinal study could provide insights into how psychological features and their impacts evolve over time with the progression of SSc, offering a deeper understanding of the long-term effects of the disease.

On the other hand, the present study has significant strengths. It investigated for the first time specific psychological wellbeing dimensions in patients with a rare disease such as SSc, providing insights and implications for both research and clinical practice. In addition, psychological features which make unique contributions to the overall status of having the diagnosis of SSc were for the first time explored.

The present study encourages to apply a multidisciplinary approach to assess and follow-up patients with systemic sclerosis since specific psychological features are worth of investigation. They may also become potential target of intervention under the global view, which is strongly accepted for this diagnosis, of limiting the severity of the non-lethal complications, either physical, mental, or psychological. Further research is of course needed.

## Data Availability

The raw data supporting the conclusions of this article will be made available by the authors, without undue reservation.
