# Supplementary material for: Psychological features of systemic sclerosis: results from an observational study
Source: Front Med (Lausanne). 2024 Dec 6;11:1473587. doi: 10.3389/fmed.2024.1473587 (PMC11658978; doi:10.3389/fmed.2024.1473587)
Supplement: Supplementary file 1 [file Table_1.docx]

**Supplementary Material.** Stepwise Multiple Regression Analysis with health status (i.e., belonging to the systemic sclerosis patient group vs healthy group) as dependent variable

| **Models** | **Dependent variables** | **df** | **β** | ***SE*** | **Z value** | **OR** | **95% LCI** | **95% UCI** | ***p*** | ***R*^2^** | ***Adjusted R*^2^** |
| --- | --- | --- | --- | --- | --- | --- | --- | --- | --- | --- | --- |
| **Model 1** | DSM-5 diagnoses | 282 | 0.262 | 0.385 | 0.679 | 1.299 | 0.614 | 2.802 | 0.500 | 0.243 | 0.148 |
|  | HAQ-DI total score | 282 | 3.276 | 0.626 | 5.231 | 26.481 | 8.461 | 99.2213 | <0.001 |  |  |
|  | PWB autonomy | 282 | -0.010 | 0.020 | 0.471 | 0.990 | 0.951 | 1.031 | 0.638 |  |  |
|  | PWB environmental mastery | 282 | 0.023 | 0.025 | 0.930 | 1.024 | 0.975 | 1.076 | 0.352 |  |  |
|  | PWB personal growth | 282 | -0.031 | 0.021 | 1.433 | 0.970 | 0.930 | 1.011 | 0.152 |  |  |
|  | PWB positive relationships with others | 282 | -0.034 | 0.02 | 1.743 | 0.966 | 0.929 | 1.004 | 0.081 |  |  |
|  | PWB purposes in life | 282 | 0.043 | 0.025 | 1.739 | 1.044 | 0.995 | 1.097 | 0.082 |  |  |
|  | PWB self-acceptance | 282 | -0.022 | 0.022 | -1.010 | 0.978 | 0.937 | 1.021 | 0.312 |  |  |
|  | SCL-90-R somatization | 282 | 0.566 | 0.491 | 1.153 | 1.762 | 0.687 | 4.761 | 0.249 |  |  |
|  | SCL-90-R obsessive-compulsive | 282 | -0.952 | 0.581 | -1.638 | 0.386 | 0.122 | 1.207 | .101 |  |  |
|  | SCL-90-R interpersonal sensibility | 282 | -0.749 | 0.703 | -1.065 | 0.473 | 0.115 | 1.851 | .287 |  |  |
|  | SCL-90-R depression | 282 | 0.623 | 0.669 | 0.932 | 1.865 | 0.508 | 7.088 | .351 |  |  |
|  | SCL-90-R anxiety | 282 | -0.703 | 0.726 | -0.968 | 0.495 | 0.119 | 2.154 | .333 |  |  |
|  | SCL-90-R hostility | 282 | 0.294 | 0.553 | 0.531 | 1.342 | 0.451 | 4.044 | .596 |  |  |
|  | SCL-90-R phobic anxiety | 282 | 0.132 | 0.780 | 0.170 | 1.141 | 0.258 | 5.641 | 0.865 |  |  |
|  | SCL-90-R paranoid ideation | 282 | -0.866 | 0.548 | -1.582 | 0.421 | 0.141 | 1.217 | 0.114 |  |  |
|  | SCL-90-R psychoticism | 282 | 1.413 | 0.800 | 1.766 | 4.110 | 0.892 | 21.167 | 0.077 |  |  |
| **Model 2** | HAQ-DI total score | 283 | 3.310 | 0.625 | 5.298 | 27.393 | 8.775 | 102.306 | <0.001 | 0.241 | 0.152 |
|  | PWB autonomy | 283 | -0.010 | 0.020 | -0.483 | 0.990 | 0.951 | 1.031 | 0.629 |  |  |
|  | PWB environmental mastery | 283 | 0.025 | 0.025 | 0.972 | 1.025 | 0.976 | 1.078 | 0.331 |  |  |
|  | PWB personal growth | 283 | -0.030 | 0.021 | -1.390 | 0.971 | 0.930 | 1.012 | 0.165 |  |  |
|  | PWB positive relationships with others | 283 | -0.034 | 0.020 | -1.753 | 0.966 | 0.929 | 1.004 | 0.080 |  |  |
|  | PWB purposes in life | 283 | 0.044 | 0.025 | 1.762 | 1.045 | 0.996 | 1.097 | 0.078 |  |  |
|  | PWB self-acceptance | 283 | -0.024 | 0.021 | -1.113 | 0.976 | 0.936 | 1.018 | 0.266 |  |  |
|  | SCL-90-R somatization | 283 | 0.547 | 0.488 | 1.121 | 1.728 | 0.677 | 4.643 | 0.262 |  |  |
|  | SCL-90-R obsessive-compulsive | 283 | -0.949 | 0.581 | -1.633 | 0.387 | 0.123 | 1.212 | 0.103 |  |  |
|  | SCL-90-R interpersonal sensibility | 283 | -0.786 | 0.703 | -1.118 | 0.456 | 0.111 | 1.784 | 0.264 |  |  |
|  | SCL-90-R depression | 283 | 0.685 | 0.662 | 1.035 | 1.984 | 0.548 | 7.452 | 0.301 |  |  |
|  | SCL-90-R anxiety | 283 | -0.660 | 0.727 | -0.907 | 0.517 | 0.125 | 2.257 | 0.364 |  |  |
|  | SCL-90-R hostility | 283 | 0.282 | 0.553 | 0.510 | 1.326 | 0.446 | 4.003 | 0.610 |  |  |
|  | SCL-90-R phobic anxiety | 283 | 0.178 | 0.774 | 0.230 | 1.195 | 0.274 | 5.842 | 0.818 |  |  |
|  | SCL-90-R paranoid ideation | 283 | -0.838 | 0.544 | -1.540 | 0.433 | 0.146 | 1.245 | 0.124 |  |  |
|  | SCL-90-R psychoticism | 283 | 1.400 | 0.798 | 1.753 | 4.049 | 0.883 | 20.768 | 0.080 |  |  |
| **Model 3** | HAQ-DI total score | 284 | 3.304 | 0.623 | 5.300 | 27.231 | 8.745 | 101.424 | <0.001 | 0.241 | 0.157 |
|  | PWB autonomy | 284 | -0.009 | 0.020 | -0.461 | 0.991 | 0.952 | 1.031 | 0.645 |  |  |
|  | PWB environmental mastery | 284 | 0.024 | 0.025 | 0.971 | 1.025 | 0.976 | 1.077 | 0.332 |  |  |
|  | PWB personal growth | 284 | -0.030 | 0.021 | -1.412 | 0.970 | 0.930 | 1.012 | 0.158 |  |  |
|  | PWB positive relationships with others | 284 | -0.035 | 0.020 | -1.761 | 0.966 | 0.929 | 1.004 | 0.078 |  |  |
|  | PWB purposes in life | 284 | 0.044 | 0.025 | 1.767 | 1.045 | 0.996 | 1.097 | 0.077 |  |  |
|  | PWB self-acceptance | 284 | -0.024 | 0.021 | -1.116 | 0.976 | 0.936 | 1.018 | 0.264 |  |  |
|  | SCL-90-R somatization | 284 | 0.564 | 0.484 | 1.164 | 1.757 | 0.693 | 4.673 | 0.244 |  |  |
|  | SCL-90-R obsessive-compulsive | 284 | -0.950 | 0.581 | -1.634 | 0.387 | 0.123 | 1.211 | 0.102 |  |  |
|  | SCL-90-R interpersonal sensibility | 284 | -0.753 | 0.689 | -1.093 | 0.471 | 0.118 | 1.787 | 0.275 |  |  |
|  | SCL-90-R depression | 284 | 0.683 | 0.660 | 1.034 | 1.979 | 0.549 | 7.390 | 0.301 |  |  |
|  | SCL-90-R anxiety | 284 | -0.618 | 0.699 | -0.884 | 0.539 | 0.141 | 2.275 | 0.377 |  |  |
|  | SCL-90-R phobic anxiety | 284 | 0.278 | 0.554 | 0.502 | 1.320 | 0.444 | 3.988 | 0.616 |  |  |
|  | SCL-90-R paranoid ideation | 284 | -0.849 | 0.542 | -1.566 | 0.428 | 0.145 | 1.227 | 0.118 |  |  |
|  | SCL-90-R psychoticism | 284 | 1.435 | 0.780 | 1.832 | 4.198 | 0.947 | 20.919 | 0.067 |  |  |
| **Model 4** | HAQ-DI total score | 285 | 3.295 | 0.623 | 5.285 | 26.968 | 8.659 | 100.361 | <0.001 | 0.241 | 0.162 |
|  | PWB autonomy | 285 | -0.009 | 0.020 | -0.427 | 0.991 | 0.952 | 1.032 | 0.670 |  |  |
|  | PWB environmental mastery | 285 | 0.024 | 0.025 | 0.938 | 1.0239 | 0.975 | 1.076 | 0.348 |  |  |
|  | PWB personal growth | 285 | -0.030 | 0.021 | -1.394 | 0.971 | 0.931 | 1.012 | 0.163 |  |  |
|  | PWB positive relationships with others | 285 | -0.034 | 0.020 | -1.749 | 0.966 | 0.929 | 1.004 | 0.080 |  |  |
|  | PWB purposes in life | 285 | 0.043 | 0.025 | 1.757 | 1.044 | 0.995 | 1.097 | 0.079 |  |  |
|  | PWB self-acceptance | 285 | -0.024 | 0.021 | -1.117 | 0.976 | 0.936 | 1.018 | 0.264 |  |  |
|  | SCL-90-R somatization | 285 | 0.527 | 0.478 | 1.102 | 1.694 | 0.675 | 4.456 | 0.270 |  |  |
|  | SCL-90-R obsessive-compulsive | 285 | -0.946 | 0.582 | -1.626 | 0.388 | 0.123 | 1.217 | 0.104 |  |  |
|  | SCL-90-R interpersonal sensibility | 285 | -0.705 | 0.679 | -1.038 | 0.494 | 0.127 | 1.845 | 0.299 |  |  |
|  | SCL-90-R depression | 285 | 0.761 | 0.642 | 1.187 | 2.141 | 0.615 | 7.718 | 0.235 |  |  |
|  | SCL-90-R anxiety | 285 | 0.502 | 0.672 | -0.747 | 0.605 | 0.165 | 2.361 | 0.455 |  |  |
|  | SCL-90-R paranoid ideation | 285 | -0.842 | 0.542 | -1.555 | 0.431 | 0.146 | 1.234 | 0.120 |  |  |
|  | SCL-90-R psychoticism | 285 | 1.367 | 0.767 | 1.783 | 3.924 | 0.909 | 18.833 | 0.075 |  |  |
| **Model 5** | HAQ-DI total score | 286 | 3.288 | 0.620 | 5.302 | 26.802 | 8.654 | 99.093 | <0.001 | 0.239 | 0.166 |
|  | PWB autonomy | 286 | -0.009 | 0.020 | -0.440 | 0.991 | 0.952 | 1.031 | 0.660 |  |  |
|  | PWB environmental mastery | 286 | 0.0267 | 0.025 | 1.075 | 1.027 | 0.979 | 1.079 | 0.282 |  |  |
|  | PWB personal growth | 286 | -0.028 | 0.021 | -1.332 | 0.972 | 0.932 | 1.013 | 0.183 |  |  |
|  | PWB positive relationships with others | 286 | -0.036 | 0.019 | -1.841 | 0.965 | 0.928 | 1.001 | 0.065 |  |  |
|  | PWB purposes in life | 286 | 0.041 | 0.024 | 1.694 | 1.042 | 0.994 | 1.094 | 0.090 |  |  |
|  | PWB self-acceptance | 286 | -0.02 | 0.021 | -1.156 | 0.975 | 0.935 | 1.017 | 0.248 |  |  |
|  | SCL-90-R somatization | 286 | 0.452 | 0.466 | 0.970 | 1.571 | 0.641 | 4.021 | 0.332 |  |  |
|  | SCL-90-R obsessive-compulsive | 286 | -1.020 | 0.573 | -1.780 | 0.361 | 0.116 | 1.110 | 0.075 |  |  |
|  | SCL-90-R depression | 286 | 0.660 | 0.626 | 1.053 | 1.934 | 0.573 | 6.771 | 0.292 |  |  |
|  | SCL-90-R psychoticism | 286 | 1.213 | 0.732 | 1.658 | 3.365 | 0.829 | 14.967 | 0.097 |  |  |
| **Model 6** | HAQ-DI total score | 287 | 3.514 | 0.585 | 6.005 | 33.578 | 11.626 | 115.940 | <0.001 | 0.237 | 0.169 |
|  | PWB autonomy | 287 | -0.007 | 0.020 | -0.324 | 0.994 | 0.955 | 1.033 | 0.746 |  |  |
|  | PWB environmental mastery | 287 | 0.026 | 0.025 | 1.071 | 1.027 | 0.979 | 1.078 | 0.284 |  |  |
|  | PWB personal growth | 287 | -0.030 | 0.021 | -1.399 | 0.970 | 0.931 | 1.012 | 0.162 |  |  |
|  | PWB positive relationships with others | 287 | -0.035 | 0.019 | -1.823 | 0.965 | 0.929 | 1.002 | 0.068 |  |  |
|  | PWB purposes in life | 287 | 0.039 | 0.024 | 1.618 | 1.040 | 0.992 | 1.091 | 0.106 |  |  |
|  | PWB self-acceptance | 287 | -0.022 | 0.021 | -1.050 | 0.978 | 0.937 | 1.020 | 0.294 |  |  |
|  | SCL-90-R obsessive-compulsive | 287 | -0.879 | 0.555 | -1.584 | 0.415 | 0.139 | 1.235 | 0.113 |  |  |
|  | SCL-90-R interpersonal sensibility | 287 | -0.752 | 0.669 | -1.126 | 0.471 | 0.123 | 1.718 | 0.260 |  |  |
|  | SCL-90-R depression | 287 | 0.840 | 0.600 | 1.396 | 2.316 | 0.722 | 7.727 | 0.163 |  |  |
|  | SCL-90-R paranoid ideation | 287 | -0.919 | 0.531 | -1.732 | 0.399 | 0.138 | 1.118 | 0.083 |  |  |
|  | SCL-90-R psychoticism | 287 | 1.214 | 0.728 | 1.667 | 3.366 | 0.837 | 14.889 | 0.096 |  |  |
| **Model 7** | HAQ-DI total score | 288 | 3.523 | 0.582 | 6.047 | 33.872 | 11.787 | 116.365 | <0.001 | 0.233 | 0.170 |
|  | PWB autonomy | 288 | -0.002 | 0.020 | -0.125 | 0.998 | 0.960 | 1.037 | 0.901 |  |  |
|  | PWB environmental mastery | 288 | 0.027 | 0.025 | 1.082 | 1.027 | 0.979 | 1.078 | 0.279 |  |  |
|  | PWB personal growth | 288 | -0.031 | 0.021 | -1.473 | 0.969 | 0.930 | 1.010 | 0.141 |  |  |
|  | PWB positive relationships with others | 288 | -0.035 | 0.019 | -1.794 | 0.966 | 0.929 | 1.003 | 0.073 |  |  |
|  | PWB purposes in life | 288 | 0.037 | 0.024 | 1.548 | 1.038 | 0.990 | 1.089 | 0.122 |  |  |
|  | PWB self-acceptance | 288 | -0.021 | 0.021 | -1.011 | 0.979 | 0.939 | 1.020 | 0.312 |  |  |
|  | SCL-90-R obsessive-compulsive | 288 | -0.932 | 0.547 | -1.703 | 0.393 | 0.133 | 1.151 | 0.090 |  |  |
|  | SCL-90-R depression | 288 | 0.716 | 0.588 | 1.217 | 2.047 | 0.652 | 6.626 | 0.223 |  |  |
|  | SCL-90-R paranoid ideation | 288 | -1.225 | 0.457 | -2.681 | 0.294 | 0.117 | 0.705 | 0.007 |  |  |
|  | SCL-90-R psychoticism | 288 | 0.961 | 0.675 | 1.422 | 2.613 | 0.708 | 10.337 | 0.155 |  |  |
| **Model 8** | HAQ-DI total score | 289 | 3.519 | 0.580 | 6.050 | 33.774 | 11.767 | 115.845 | <0.001 | 0.233 | 0.176 |
|  | PWB environmental mastery | 289 | 0.026 | 0.020 | 1.080 | 1.026 | 0.979 | 1.077 | 0.280 |  |  |
|  | PWB personal growth | 289 | -0.032 | 0.020 | -1.533 | 0.969 | 0.930 | 1.009 | 0.125 |  |  |
|  | PWB positive relationships with others | 289 | -0.035 | 0.020 | -1.815 | 0.966 | 0.929 | 1.002 | 0.070 |  |  |
|  | PWB purposes in life | 289 | 0.038 | 0.024 | 1.571 | 1.038 | 0.991 | 1.089 | 0.116 |  |  |
|  | PWB self-acceptance | 289 | -0.022 | 0.020 | -1.087 | 0.978 | 0.940 | 1.018 | 0.277 |  |  |
|  | SCL-90-R obsessive-compulsive | 289 | -0.931 | 0.547 | -1.702 | 0.394 | 0.134 | 1.152 | 0.089 |  |  |
|  | SCL-90-R depression | 289 | 0.714 | 0.588 | 1.213 | 2.042 | 0.651 | 6.612 | 0.225 |  |  |
|  | SCL-90-R paranoid ideation | 289 | -1.225 | 0.457 | -2.684 | 0.294 | 0.117 | 0.704 | 0.007 |  |  |
|  | SCL-90-R psychoticism | 289 | 0.959 | 0.675 | 1.420 | 2.609 | 0.707 | 10.317 | 0.156 |  |  |
| **Model 9** | HAQ-DI total score | 290 | 3.500 | 0.582 | 6.016 | 33.114 | 11.539 | 113.531 | <0.001 | 0.230 | 0.178 |
|  | PWB personal growth | 290 | -0.030 | 0.020 | -1.460 | 0.971 | 0.932 | 1.010 | 0.144 |  |  |
|  | PWB positive relationships with others | 290 | -0.031 | 0.018 | -1.647 | 0.970 | 0.934 | 1.006 | 0.01 |  |  |
|  | PWB purposes in life | 290 | 0.046 | 0.022 | 2.017 | 1.047 | 1.002 | 1.09 | 0.044 |  |  |
|  | PWB self-acceptance | 290 | -0.014 | 0.019 | -0.753 | 0.986 | 0.949 | 1.023 | 0.451 |  |  |
|  | SCL-90-R obsessive-compulsive | 290 | 0.989 | 0.541 | -1.829 | 0.372 | 0.128 | 1.072 | 0.067 |  |  |
|  | SCL-90-R depression | 290 | 0.649 | 0.584 | 1.110 | 1.913 | 0.614 | 6.139 | 0.267 |  |  |
|  | SCL-90-R paranoid ideation | 290 | -1.240 | 0.458 | -2.630 | 0.300 | 0.119 | 0.721 | 0.007 |  |  |
|  | SCL-90-R psychoticism | 290 | 0.991 | 0.672 | 1.475 | 2.695 | 0.739 | 10.634 | 0.140 |  |  |
| **Model 10** | HAQ-DI total score | 291 | 3.461 | 0.577 | 6.001 | 31.852 | 11.200 | 108.029 | <0.001 | 0.229 | 0.181 |
|  | PWB personal growth | 291 | -0.030 | 0.020 | -1.478 | 0.970 | 0.932 | 1.010 | 0.140 |  |  |
|  | PWB positive relationships with others | 291 | -0.034 | 0.018 | -1.833 | 0.967 | 0.932 | 1.002 | 0.067 |  |  |
|  | PWB purposes in life | 291 | 0.038 | 0.020 | 1.881 | 1.039 | 0.999 | 1.080 | 0.060 |  |  |
|  | SCL-90-R obsessive-compulsive | 291 | -0.989 | 0.542 | -1.823 | 0.372 | 0.127 | 1.075 | 0.068 |  |  |
|  | SCL-90-R depression | 291 | 0.749 | 0.570 | 1.313 | 2.115 | 0.697 | 6.600 | 0.189 |  |  |
|  | SCL-90-R paranoid ideation | 291 | -1.219 | 0.455 | -2.683 | 0.295 | 0.118 | 0.705 | 0.007 |  |  |
|  | SCL-90-R psychoticism | 291 | 1.006 | 0.672 | 1.496 | 2.735 | 0.749 | 10.822 | 0.135 |  |  |
| **Model 11** | HAQ-DI total score | 292 | 3.479 | 0.578 | 6.023 | 32.421 | 11.377 | 110.127 | <0.001 | 0.228 | 0.182 |
|  | PWB personal growth | 292 | -0.033 | 0.020 | -1.608 | 0.968 | 0.930 | 1.007 | 0.108 |  |  |
|  | PWB positive relationships with others | 292 | -0.031 | 0.018 | -1.716 | 0.969 | 0.935 | 1.004 | 0.086 |  |  |
|  | PWB purposes in life | 292 | 0.032 | 0.020 | 1.633 | 1.033 | 0.994 | 1.075 | 0.102 |  |  |
|  | SCL-90-R obsessive-compulsive | 292 | -0.538 | 0.420 | -1.282 | 0.584 | 0.255 | 1.332 | 0.200 |  |  |
|  | SCL-90-R paranoid ideation | 292 | -1.189 | 0.453 | -2.620 | 0.305 | 0.122 | 0.725 | 0.009 |  |  |
|  | SCL-90-R psychoticism | 292 | 1.270 | 0.645 | 1.969 | 3.562 | 1.038 | 13.432 | 0.049 |  |  |
| **Model 12** | HAQ-DI total score | 293 | 3.397 | 0.572 | 5.932 | 29.871 | 10.584 | 100.532 | 0.001 | 0.224 | 0.183 |
|  | PWB personal growth | 293 | -0.034 | 0.020 | -1.687 | 0.966 | 0.928 | 1.005 | 0.092 |  |  |
|  | PWB positive relationships with others | 293 | -0.029 | 0.018 | -1.616 | 0.971 | 0.937 | 1.006 | 0.106 |  |  |
|  | PWB purposes in life | 293 | 0.034 | 0.020 | 1.737 | 1.035 | 0.100 | 1.070 | 0.082 |  |  |
|  | SCL-90-R paranoid ideation | 293 | -1.373 | 0.430 | -3.196 | 0.253 | 0.106 | 0.574 | 0.001 |  |  |
|  | SCL-90-R psychoticism | 293 | 0.943 | 0.587 | 1.607 | 2.569 | 0.834 | 8.542 | 0.108 |  |  |
| **Model 13** | HAQ-DI total score | 295 | 3.376 | 0.571 | 5.913 | 29.261 | 10.411 | 98.322 | <0.001 | 0.212 | 0.181 |
|  | PWB personal growth | 295 | -0.032 | 0.020 | -1.574 | 0.969 | 0.931 | 1.008 | 0.116 |  |  |
|  | PWB positive relationships with others | 295 | -0.033 | 0.018 | -1.858 | 0.967 | 0.934 | 1.002 | 0.063 |  |  |
|  | PWB purposes in life | 295 | 0.028 | 0.019 | 1.447 | 1.028 | 0.990 | 1.069 | 0.148 |  |  |
|  | SCL-90-R paranoid ideation | 295 | -1.038 | 0.366 | -2.834 | 0.354 | 0.169 | 0.716 | 0.005 |  |  |
| **Model 14** | HAQ-DI total score | 295 | 3.252 | 0.559 | 5.818 | 25.843 | 9.408 | 84.792 | <0.001 | 0.207 | 0.181 |
|  | PWB personal growth | 295 | -0.022 | 0.019 | -1.146 | 0.979 | 0.943 | 1.015 | 0.252 |  |  |
|  | PWB positive relationships with other | 295 | -0.023 | 0.016 | -1.414 | 0.977 | 0.946 | 1.009 | 0.157 |  |  |
|  | SCL-90-R paranoid ideation | 295 | -1.104 | 0.362 | -3.044 | 0.331 | 0.159 | 0.665 | 0.002 |  |  |

*Note.* β = regression coefficient. SE = Standard Error. Z = ratio of the estimate to its standard error. OR = Odds Ratio. 95%LCI = lower value of the 95% confidence interval. 95%UCI = upper value of the 95% confidence interval. *R*² = McFaden Pseudo R

DSM-5: Diagnostic and Statistical Manual of mental disorders. HAQ-DI: Health Assessment Questionnaire Disability Index. PWB: Psychological Well Being Scales. SCL-90-R: Symptom Checklist-90-Revised
